# Supplementary material for: Online pragmatic interpretations of scalar adjectives are affected by perceived speaker reliability
Source: PLoS One. 2021 Feb 19;16(2):e0245130. doi: 10.1371/journal.pone.0245130 (PMC7895354; doi:10.1371/journal.pone.0245130)
Supplement: S1 Appendix — (DOCX) [file pone.0245130.s001.docx]

**S1 Appendix: Explicit instructions in the reliable- and unreliable-speaker conditions**

[The initial and final parts were identical across the two between-participant conditions]

“In this experiment you will be asked to select objects according to a set of spoken instructions. Each trial will begin with four objects arranged in a grid on the screen in front of you. The instructions were recorded by a person who was in the experiment before you. He saw all the same objects that you will see and was asked to give instructions that would direct a listener throughout the experiment. Let's take a look at a few examples.”

[Here we play a video clip]

**Reliable-speaker**: “The study is intended to measure how effectively people communicate in various situations. In order to evaluate how well the speaker conveyed the intended meaning, it is important for you to respond as accurately and quickly as you can. Because the speaker didn't have much time to practice, some of the instructions may be confusing or silly.”

**Unreliable-speaker**: “The study is intended to examine communicative aspects of his language impairment. In order to evaluate how well the speaker conveyed the intended meaning, it is important for you to respond as accurately and quickly as you can. Because of the speaker's impairment, some of the instructions may sound odd or confusing.”

“Please try the best you can. If you have any questions, please ask the experimenter now.”
