# Supplementary material for: Online pragmatic interpretations of scalar adjectives are affected by perceived speaker reliability
Source: PLoS One. 2021 Feb 19;16(2):e0245130. doi: 10.1371/journal.pone.0245130 (PMC7895354; doi:10.1371/journal.pone.0245130)
Supplement: S2 Appendix — (DOCX) [file pone.0245130.s002.docx]

**S2 Appendix: Data variations across participants and items**


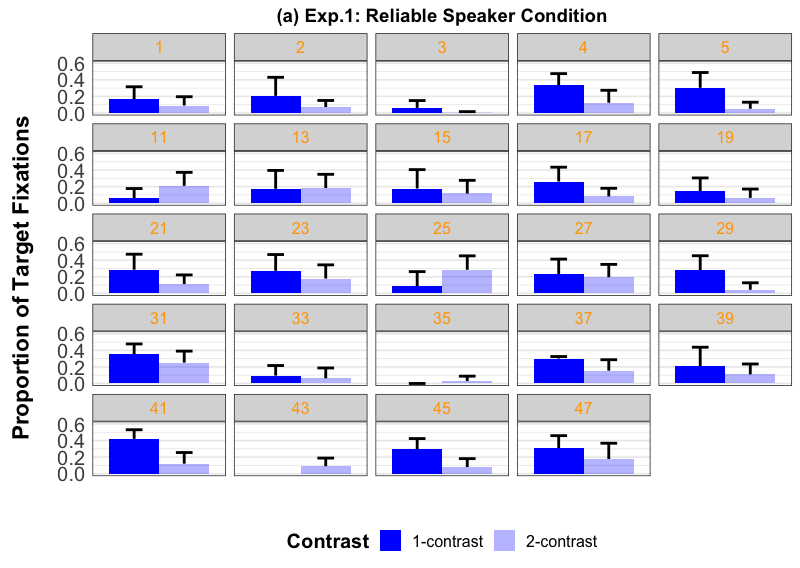


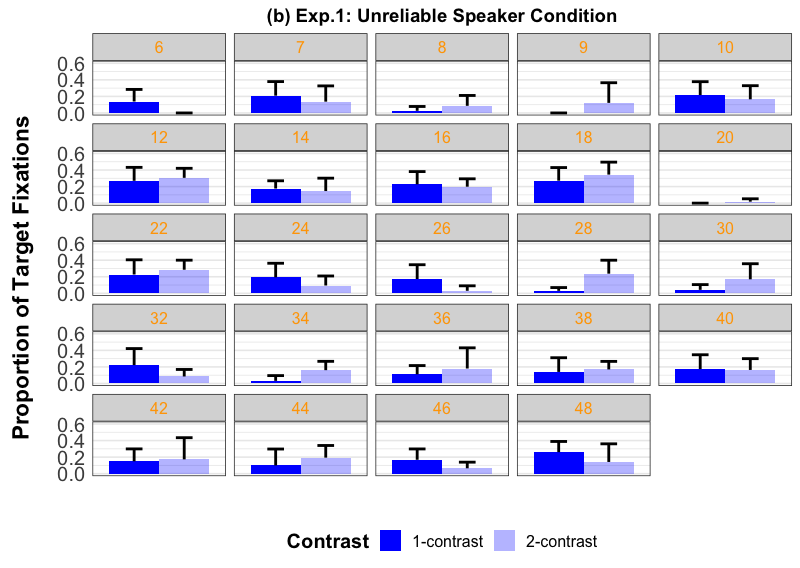


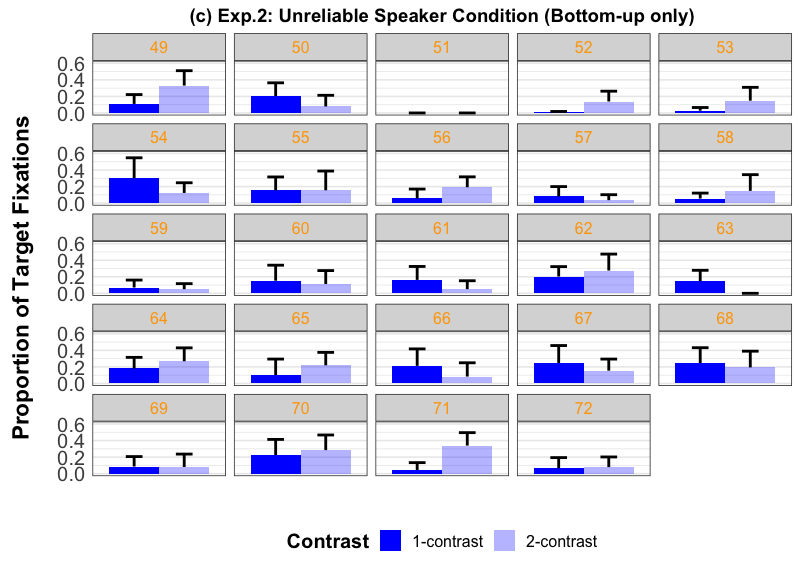


Fig 1. Proportion of target fixations by subjects. (1) and (2) show the reliable- and unreliable-speaker conditions in Experiment 1, respectively. (3) shows the unreliable-speaker condition in Experiment 2 (bottom-up information only).


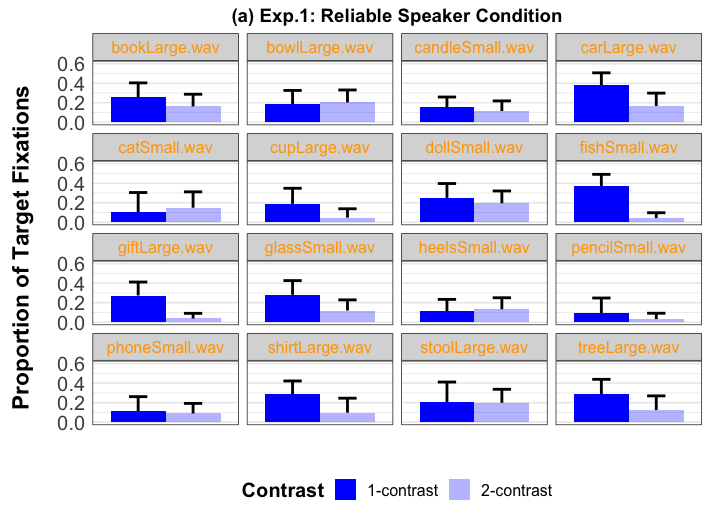


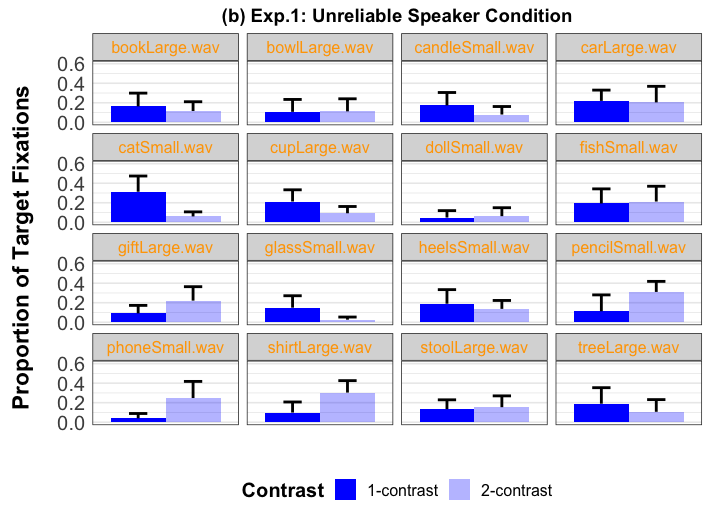


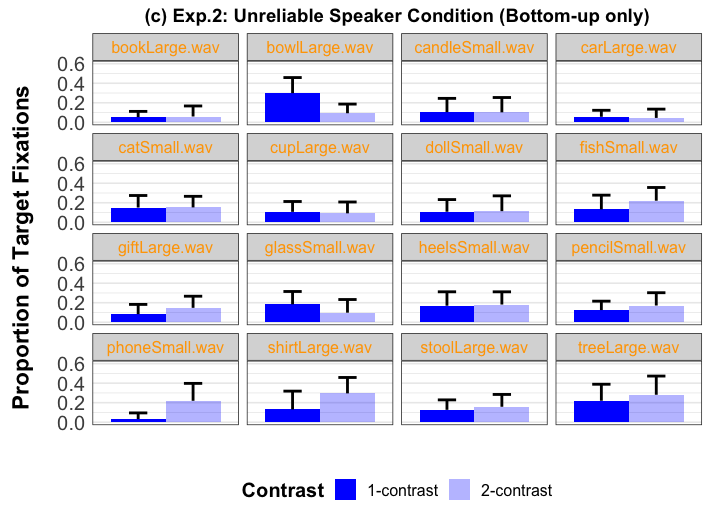


Fig 2. Proportion of target fixations by items. (1) and (2) show the reliable- and unreliable-speaker conditions in Experiment 1, respectively. (3) shows the unreliable-speaker condition in Experiment 2 (bottom-up information only).
