# Supplementary material for: Online pragmatic interpretations of scalar adjectives are affected by perceived speaker reliability
Source: PLoS One. 2021 Feb 19;16(2):e0245130. doi: 10.1371/journal.pone.0245130 (PMC7895354; doi:10.1371/journal.pone.0245130)
Supplement: S3 Appendix — (DOCX) [file pone.0245130.s003.docx]

**S3 Appendix: Post-hoc analyses of fixation proportions using Generalized Additive Mixed Effect Models (GAMMs)**

To investigate the effect of the order of items, we used Generalized Additive Mixed Models (GAMMs). GAMMs employ smooth functions to model non-linear effects and allow inclusion of random effects to adequately account for the repeated measures structure of our data. GAMMs are widely used to analyze psychophysical data and previously adopted to examine eye-tracking data e.g., (87). Three GAMMs with binomial (logit) link function were fit separately to the target fixations for the three reliability conditions (weighted, following (49)). Each GAMM included contrast (sum-coded as in the logistic regression models reported in Experiments 1 and 2), item order (i.e., critical items 1 to 16), and time relative to the adjective onset, as well as interactions between them. Specifically, the interaction of time and item order were modeled as separate tensor product smooths for each of the two levels of contrast. Additionally, the GAMM contained random by-item intercepts and factor smooths for time, as well as random by-participant intercepts and factor smooths for the time, item order, and their interaction. The goal here was to explore changes in target fixations in response to an adjective *throughout the experiment*.

Fig 1 shows the target fixations (indicated through color) for the 1-contrast condition (left), 2-contrast condition (middle), as well as the difference in target fixations between the two contrast conditions (right), for each of the three reliability conditions. Time within a trial since the onset of the scalar adjective is shown on the horizontal axis. Item order is shown along the vertical axis. Significant differences between the two contrast conditions are indicated by an absence of opaqueness (right panel). These significances are likely anti-conservative (because of the auto-correlations between samples from the eye-tracker) and therefore have to be interpreted with caution. For the present purpose, however, we regard them to be sufficiently informative because the degree of anti-conservativity is more or less constant across the three reliability conditions. To facilitate comparison across speaker conditions (see right-most panels of Fig 1), we highlighted areas in which the differences between the contrast conditions were larger than ten standard errors.

a) Reliable speaker condition (Experiment 1)

###

b) Unreliable speaker condition (Experiment 1, with explicit instructions)

c) Experiment 2 (unreliable, bottom-up only)

Fig 1. Topographical maps for the target fixation models a) reliable-speaker condition in Experiment 1, b) unreliable-speaker condition in Experiment 1 and c) Experiment 2. All plots: Time in relation to the adjective onset (in milliseconds) is represented on the x-axis. Item order (critical trials only) is on the y-axis. In each row: model prediction for the (i) 1-contrast condition, (ii) 2-contrast condition, and (iii) difference between the 1- and 2-contrast conditions. For (i) and (ii), estimated color and contour lines indicate predicted proportions of target fixations. For (iii) color and contour lines indicate the predicted difference in log-odds; lack of opaqueness indicates large differences (see text).
