# Supplementary material for: Online pragmatic interpretations of scalar adjectives are affected by perceived speaker reliability
Source: PLoS One. 2021 Feb 19;16(2):e0245130. doi: 10.1371/journal.pone.0245130 (PMC7895354; doi:10.1371/journal.pone.0245130)
Supplement: S4 Appendix — (DOCX) [file pone.0245130.s004.docx]

**S4 Appendix: Mouse-clicking response times**

To gain further insight into the effect of the reliability manipulations, we analyzed the mouse-clicking response times (i.e., the duration between sentence onset and the time when a picture is clicked in each trial). The mean response times across the three conditions are summarized in Table 1. Distributions of response times by reliability and contrast conditions are presented in Fig 1.

Table 1. Mean response times in Experiments 1 and 2.

| Reliability | Mean (ms) | Standard deviations |
| --- | --- | --- |
| reliable | 2,964 | 317 |
| unreliable (bottom-up only, Experiment 2) | 3,139 | 367 |
| unreliable (with explicit instruction, Experiment 1) | 3,106 | 307 |


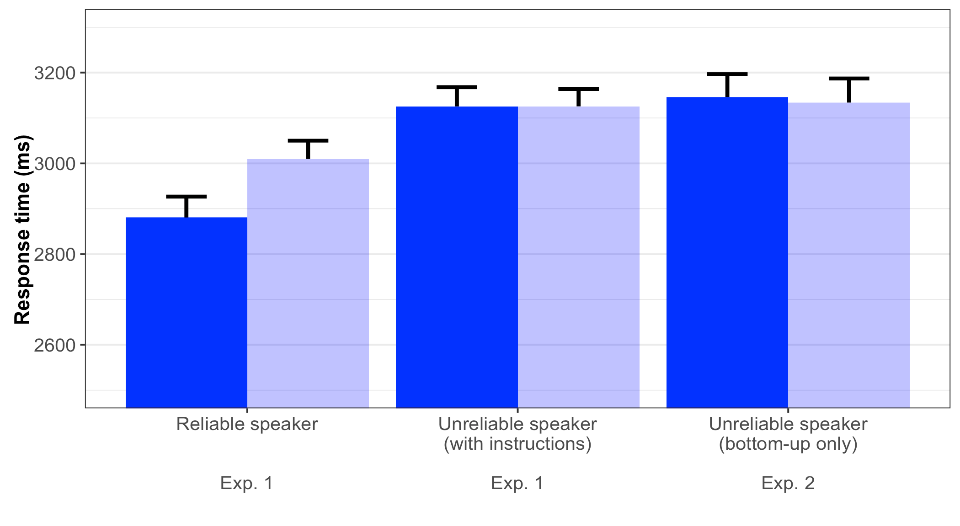

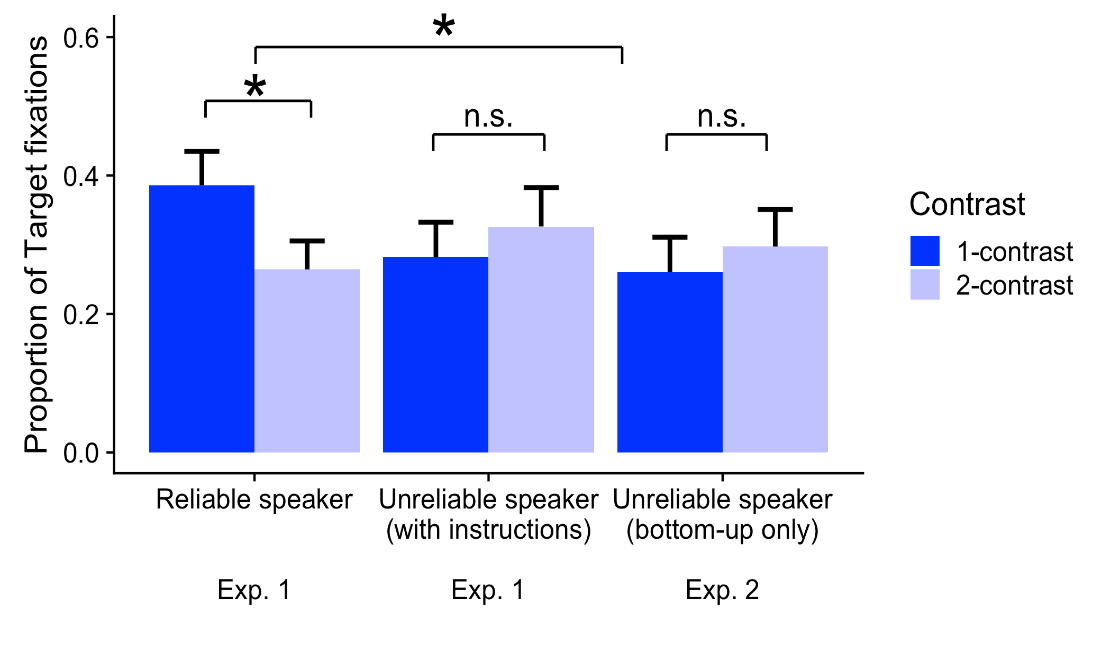


Fig 1. Mean response times (time duration between onset of an audio instruction and the time point at which a mouse-clicking response is made) by reliability and contrast conditions.

To analyze the data, we first removed observations that were three standard deviations away from the participant mean (45 instances, 3.7% of the data). Then, we conducted the following analyses to test whether the speaker-reliability and contrast manipulations affected the timing of participants’ explicit mouse-clicking responses in each trial.

To account for the general trend in which participants accelerate in their responses over the course of the experiment, we first ran an ordinary linear regression model to predict the response time with the trial number as a predictor. This model included both the critical and filler items. The trial number, as expected, was a significant predictor (*β =* -1.42, *t* = -2.18, *p* < 0.03). We then applied a mixed effect regression model to predict the residualized response time (i.e., variability of response times not accounted for by the trial order). This model included data only from the critical trials. As in the fixation proportion models reported in Experiments 1 and 2, it contained the reliability conditions (Helmert coded as described in Table 3) and contrast conditions (sum-coded: 1 = 1-contrast vs. -1 = 2-contrast). The model converged with the maximum random effect structure justified by the design. We also conducted an analysis including the item order (e.g., the order in which the critical items were presented in the experiment) as a fixed effect. Model comparison using ANOVA determined that item order and its interactions with contrast and reliability did not significantly improve the model fit.

Table 2 presents the model summary. Participants overall responded more rapidly in 1-contrast compared to 2-contrast conditions (*β=* -19.59, *t* = 2.41, *p* < 0.03). Importantly, the reliability manipulation between the reliable- and unreliable-speaker conditions was a significant predictor of the residualized response time, such that participants were faster to select a referent when the speaker was reliable (*β=* -125.12, *t* = -3.34, *p* < 0.001). The null effect of the reliability manipulation between the unreliable-speaker with explicit instructions (Experiment 1) and the unreliable-speaker with bottom-up input only (Experiment 2) suggests that participants were equally slow to respond in both unreliable-speaker conditions. There was also a significant positive interaction between the contrast and the reliability conditions (reliable vs. unreliable): participants in the reliable-speaker conditions were even faster to select a referent in the 1-contrast condition than would be expected based on the main effect of the contrast condition (*β=* -44.98, *t* = 4.46, *p* < 0.0001).

Table 2. Model summary of residualized mouse-clicking response times in Experiments 1 and 2.

| Experiments 1 and 2: Fixed effects | | | | |
| --- | --- | --- | --- | --- |
|  | *β* | Std. Error | *t*-value | *p*-value |
| (Intercept) | -176.886 | 32.485 | -5.445 | <.0001 |
| Reliability (reliable vs. unreliable) | **-125.115** | **37.457** | **-3.34** | **<.001** |
| Reliability (unreliable bottom-up only vs. unreliable with explicit instruction) | 7.224 | 32.088 | .225 | n.s. |
| Contrast (1-contrast vs. 2-contrast) | **-19.587** | **8.134** | **2.408** | **<.03** |
| Reliability (reliable vs. unreliable) * contrast (1-contrast vs. 2-contrast) | **-44.98** | **10.087** | **4.459** | **<.0001** |
| Reliability (bottom-up only vs. unreliable with explicit instruction) * contrast (1-contrast vs. 2-contrast) | 2.953 | 8.736 | .338 | n.s. |
| Number of observations: 1107; Participants: 72; Items: 16 | | | | |

The eye movements and mouse-clicking response times thus paint a coherent picture that participants in the reliable-speaker condition were overall faster to respond to the verbal instruction, particularly in the 1-contrast condition where they could generate a contrastive inference. Further, participants who received only the bottom-up input that insinuates the unreliability of the speaker (Experiment 2) modulated their response behaviors to a similar degree as those who received both the explicit characterization of the unreliability of the speaker as well as the bottom-up evidence of idiosyncratic language use (Experiment 1).
